# Supplementary material for: Wing morphological responses to latitude and colonisation in a range expanding butterfly
Source: PeerJ. 2020 Nov 19;8:e10352. doi: 10.7717/peerj.10352 (PMC7680626; doi:10.7717/peerj.10352)
Supplement: Supplemental Information 1 [file peerj-08-10352-s001.docx]

| **Site name** | **Exp ^a^** | **Latitude** | **Longitude** | **10km grid** | **Years col. ^b^** | **Collection year** | **n ^c^** |
| --- | --- | --- | --- | --- | --- | --- | --- |
| Allean Forest | S | 56.718415 | -3.871214 | NN86 | 15 | 2018 | 20 |
| Ashberry and Reins Wood | E | 54.257506 | -1.1265357 | SE58 | 12 | 2018 | 20 |
| Ashington Community Woods | E | 55.19456 | -1.583699 | NZ28 | 12 | 2018 | 20 |
| Calke Abbey | E | 52.783543 | -1.46656 | SK32 | 40 | 2018 | 20 |
| Carr Brae | S | 57.268149 | -5.503034 | NG82 | 42 | 2018 | 10 |
| Carverel Copse | E | 51.076197 | -1.7230305 | SU13 | 36 | 2016 | 20 |
| Cliburn Moss | E | 54.624693 | -2.659253 | NY52 | 10 | 2018 | 20 |
| Crathes Castle | S | 57.069417 | -2.47805 | NO79 | 10 | 2018 | 20 |
| Creag Dhomhainn | S | 57.455318 | -4.3517008 | NH54 | 42 | 2018 | 20 |
| Culzean Country Park | E | 55.34981 | -4.802654 | NS20 | 10 | 2018 | 20 |
| Dava Way | S | 57.508783 | -3.6423181 | NJ04 | 23 | 2017 | 20 |
| Drum Wood | S | 57.452008 | -3.3547838 | NJ14 | 20 | 2017 | 20 |
| Eyemouth | E | 55.856552 | -2.100613 | NT95 | 10 | 2018 | 20 |
| Fenn's Whixhall and Bettisfield Moss | E | 52.924251 | -2.7793818 | SJ43 | 46 | 2017 | 20 |
| Fermyn Woods | E | 52.458933 | -0.58119336 | SP98 | 48 | 2018 | 20 |
| Foxley Wood | E | 52.76478 | 1.037034 | TG02 | 50 | 2018 | 20 |
| Garlieston | E | 54.791978 | -4.35249 | NX44 | 6 | 2018 | 10 |
| Glasdrum Wood | S | 56.558704 | -5.2577049 | NM94 | 52 | 2017 | 20 |
| Gorebridge* | E | 55.845534 | -3.060799 | NT16 | 4 | 2018 | 30 |
| Hadleigh Railway Walk | E | 52.024835 | 0.99390923 | TM04 | 24 | 2017 | 20 |
| Haw Park Wood | E | 53.631296 | -1.451966 | SE31 | 24 | 2018 | 15 |
| Hudson Way, SE94 | E | 53.87701 | -0.61577753 | SE94 | 15 | 2017 | 6 |
| Hudson Way, SE84 | E | 53.870981 | -0.63879088 | SE84 | 15 | 2017 | 14 |
| Hutton Roof Crags | E | 54.190345 | -2.6972663 | SD57 | 26 | 2018 | 20 |
| Langley Wood | E | 54.949076 | -2.263599 | NY86 | 9 | 2018 | 10 |
| Llanymynech Hill | E | 52.795064 | -3.0885165 | SJ22 | 46 | 2017 | 10 |
| Llanymynech Rocks | E | 52.789685 | -3.0868993 | SJ22 | 46 | 2017 | 10 |
| Marshall Heath | E | 51.820673 | -0.31694514 | TL11 | 47 | 2018 | 18 |
| Martin Down NNR | E | 50.982097 | -1.951515 | SU02 | 46 | 2017 | 5 |
| Migdale Wood | S | 57.885965 | -4.249507 | NH69 | 23 | 2018 | 20 |
| Resipole Farm | S | 56.71185 | -5.719805 | NM76 | 50 | 2018 | 10 |
| Robinswood Hill Country Park | E | 51.836213 | -2.235444 | SO81 | 42 | 2018 | 20 |
| Roudsea Wood* | E | 54.235261 | -3.029904 | SD38 | 19 | 2018 | 20 |
| Ruff Wood | E | 54.235261 | -3.029904 | SD40 | 26 | 2018 | 10 |
| Shian Wood | S | 56.518689 | -5.4087614 | NM94 | 52 | 2017 | 20 |
| Snapes Wood | E | 51.111592 | -1.9828075 | SU03 | 51 | 2016 | 14 |
| Snuff Mill Lane | E | 53.772701 | -0.39615076 | TA03 | 15 | 2017 | 20 |
| Soddy Gap | E | 54.678997 | -3.4392211 | NY03 | 10 | 2018 | 20 |
| South Laggan Forest | S | 57.001156 | -4.8845325 | NN29 | 10 | 2018 | 7 |
| Stour Wood | E | 51.935411 | 1.1800119 | TM13 | 34 | 2017 | 10 |
| Taynish NNR | S | 56.001966 | -5.636926 | NR78 | 50 | 2018 | 20 |
| Walton Colliery NP | E | 53.653644 | -1.452258 | SE31 | 24 | 2018 | 15 |
| Witton Le Wear | E | 54.67831 | -1.751833 | NZ13 | 16 | 2018 | 20 |
| Wolves Wood | E | 52.053529 | 0.99728714 | TM04 | 24 | 2017 | 10 |
| Woodcock Wood | E | 50.70228 | -3.8975018 | SX69 | 44 | 2018 | 20 |
| Yellowcraig Wood | E | 56.06078 | -2.778829 | NT58 | 8 | 2018 | 20 |
|  |  |  |  |  |  | **TOTAL** | **774** |
