# Supplementary material for: Wing morphological responses to latitude and colonisation in a range expanding butterfly
Source: PeerJ. 2020 Nov 19;8:e10352. doi: 10.7717/peerj.10352 (PMC7680626; doi:10.7717/peerj.10352)
Supplement: Supplemental Information 2 [file peerj-08-10352-s002.docx]

| **Predictors** | **FOREWING** | | | | | | | | **HINDWING** | | | | | | | |
| --- | --- | --- | --- | --- | --- | --- | --- | --- | --- | --- | --- | --- | --- | --- | --- | --- |
|  | **Dorsal** | | | | **Ventral** | | | | **Dorsal** | | | | **Ventral** | | | |
|  | ***Est.*** | ***SE*** | ***df*** | ***t*** | ***Est.*** | ***SE*** | ***df*** | ***t*** | ***Est.*** | ***SE*** | ***df*** | ***t*** | ***Est.*** | ***SE*** | ***df*** | ***t*** |
| (Intercept) | 108.57 | 97.65 | 36.20 | 1.11 | 102.94 | 89.83 | 14.85 | 1.15 | 58.12 | 95.97 | 22.33 | 0.61 | -4.04 | 81.13 | 51.59 | -0.05 |
| Latitude | -0.68 | 1.63 | 31.53 | -0.42 | 0.57 | 1.46 | 7.73 | 0.39 | 0.79 | 1.58 | 14.02 | 0.50 | 2.55 | 1.14 | 47.23 | **2.24** |
| Mean 10-year annual temperature (T_10_) | 5.30 | 2.73 | 55.68 | 1.94 | 3.06 | 2.61 | 53.36 | 1.17 | 3.34 | 2.72 | 53.47 | 1.23 | 3.44 | 2.73 | 57.30 | 1.26 |
| Temperature during development | -2.45 | 1.35 | 41.23 | -1.81 | -2.79 | 1.33 | 40.12 | **-2.10** | -2.53 | 1.38 | 37.05 | -1.83 | -2.94 | 1.49 | 39.02 | **-1.97** |
| Years colonised | 0.05 | 0.14 | 32.18 | 0.38 | 0.12 | 0.13 | 8.50 | 0.91 | 0.05 | 0.14 | 15.16 | 0.33 | 0.09 | 0.11 | 37.72 | 0.90 |
| **Random Effects** | | | | | | | | | | | | | | | | |
| σ^2^ | 109.94 | | | | 98.47 | | | | 100.51 | | | | 85.77 | | | |
| τ_00_ | 48.07 _Julian day difference_ | | | | 35.43 _Julian day difference_ | | | | 36.54 _Julian day difference_ | | | | 24.06 _Julian day difference_ | | | |
|  | 54.23 _Grid.10km:Expansion_ | | | | 58.75 _Grid.10km:Expansion_ | | | | 64.16 _Grid.10km:Expansion_ | | | | 90.58 _Grid.10km:Expansion_ | | | |
|  | 183.10 _Expansion_ | | | | 51.47 _Expansion_ | | | | 85.30 _Expansion_ | | | | 0.00 _Expansion_ | | | |
| ICC | 0.72 | | | | 0.60 | | | | 0.65 | | | | 0.57 | | | |
| N | 43 _Grid.10km_ | | | | 43 _Grid.10km_ | | | | 43 _Grid. 10km_ | | | | 43 _Grid. 10km_ | | | |
|  | 2 _Expansion_ | | | | 2 _Expansion_ | | | | 2 _Expansion_ | | | | 2 _Expansion_ | | | |
|  | 53 _Julian day difference_ | | | | 53 _Julian day difference_ | | | | 53 _Julian day difference_ | | | | 53 _Julian day difference_ | | | |
| Observations | 709 | | | | 709 | | | | 642 | | | | 641 | | | |
| Marginal R^2^ / Conditional R^2^ | 0.06 / 0.74 | | | | 0.06 / 0.62 | | | | 0.04 / 0.66 | | | | 0.16 / 0.64 | | | |
| Significant *t* values with a magnitude greater than 1.96 are indicated in bold. σ^2^= Residual variance; τ_00_ = Random effect variance; ICC= Interclass correlation coefficient | | | | | | | | | | | | | | | | |
