# Supplementary figures and images for: Wing morphological responses to latitude and colonisation in a range expanding butterfly
Source: PeerJ. 2020 Nov 19;8:e10352. doi: 10.7717/peerj.10352 (PMC7680626; doi:10.7717/peerj.10352)

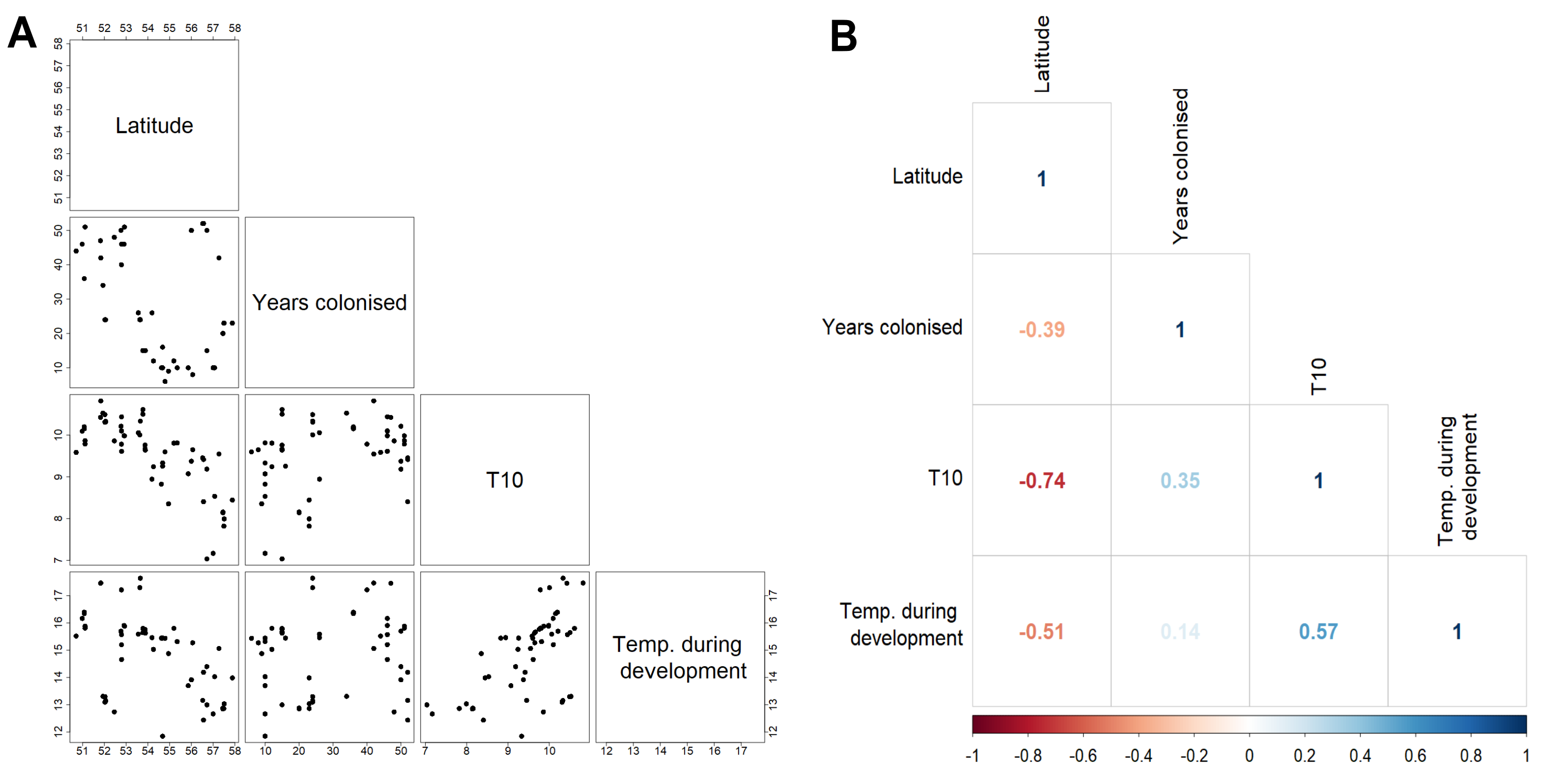

Supplement: Supplemental Information 3 — Correlation scatterplots (A) and the Pearson correlation coefficient (B) for the environmental and demographic variables used in the linear mixed models. [file peerj-08-10352-s003.png]
